# Dog Park Membership and Life Satisfaction Among Older Adults

**DOI:** 10.1093/geroni/igab046.3140

**Published:** 2021-12-17

**Authors:** Betz King, Adam Duberstein, McGlinn Maureen

**Affiliations:** 1 King & Associates, King & Associates, Michigan, United States; 2 King & Associates, Hazel Park, Michigan, United States; 3 Southfield Mental Health, Southfield, Michigan, United States

## Abstract

Dog park members initially join and attend dog parks for the wellbeing of their dogs, but often experience their own biopsychosocial benefits. This mixed methodology (Quantitative n=44, Qualitative n=11) ) pilot study utilized qualitative heuristic interviewing (Moustakas, C., 1990) and the Satisfaction with Life Survey (Pavot, W., & Diener, E. 2013). Data gathered from interviews and surveys administered to participants of a members-only dog-park indicate a high satisfaction with life. Members 60 years and older reported feelings of life satisfaction almost 7 points over the total respondent average, placing them in the “highly satisfied” range. All members experience the dog-park as a supportive social environment that benefits their physical health, mental health and the well-being of their canine companions. Five qualitative themes were identified: Canine Well-being, Community, Mental Health Benefits, Physical Health Benefits and Fights, Falls & Frustrations. These findings demonstrate the need for more research into the impact and importance of pet ownership, community dog parks and outdoor green spaces on older adults and life satisfaction.

